# Construction of a novel radiosensitivity- and ferroptosis-associated gene signature for prognosis prediction in gliomas

**DOI:** 10.7150/jca.72893

**Published:** 2022-05-20

**Authors:** Yandong Xie, Yong Xiao, Yuyang Liu, Xueying Lu, Zhen Wang, Shuo Sun, Liang Liu, Xianglong Tang, Hong Xiao, Hongyi Liu

**Affiliations:** 1 Department of Neurosurgery, The Affiliated Brain Hospital of Nanjing Medical University, Fourth Clinical College of Nanjing Medical University, Nanjing 210029, China.; 2 Department of Hematology, The First Affiliated Hospital of Nanjing Medical University, Jiangsu Province Hospital, Nanjing 210029, China.; 3 Department of Neuro-Psychiatric Institute, The Affiliated Brain Hospital of Nanjing Medical University, Nanjing 210029, China.

**Keywords:** Gliomas, prognosis, biomarker, radiosensitivity, ferroptosis

## Abstract

**Background:** Gliomas are the most refractory intracranial disease characterized by high incidence and mortality rates. Therefore, radiotherapy plays a crucial role in the treatment of gliomas. However, recent evidence reveals that ferroptosis is highly associated with radiosensitivity in tumor cells. Therefore, this study aimed to investigate radiosensitivity- and ferroptosis-associated biomarkers. Moreover, the study aimed to provide new strategies for the treatment and evaluation of prognosis in gliomas.

**Methods:** The mRNA sequencing and relevant clinical data were obtained from The Cancer Genome Atlas (TCGA). Secondly, differential analysis was conducted to reveal the radiosensitivity- and ferroptosis-associated differentially expressed genes (DEGs). Further, a predictive model based on the seven genes was constructed, and LASSO regression analysis was carried out. After that, the Chinese Glioma Genome Atlas (CGGA) was used for validation of the results.

**Results:** A total of 36 radiosensitivity- and 19 ferroptosis-associated DEGs with a prognostic value were identified. Moreover, seven intersecting genes (HSPB1, STAT3, CA9, MAP1LC3A, MAPK1, ZEB1, and TNFAIP3) were identified as the risk signature genes. The ROC curves and K-M analysis revealed that the signature genes showed a good survival prediction. Furthermore, the functional analysis revealed that the differentially expressed genes between the high-risk and the low-risk groups were enriched in glioma-related biological processes. In addition, differences were reported in immune function status between the two groups.

**Conclusion:** This study revealed that the seven biomarkers could help predict the prognosis in glioma patients. In addition, this study provides a basis for understanding the molecular mechanisms of radiosensitivity and ferroptosis in the treatment of gliomas.

## Introduction

Gliomas are tumors originating from glial cells and are the most common malignant tumors of the central nervous system. Glioma patients have a median survival of fewer than 15 months, with a five-year survival rate of less than 5.5% 1. The incidence rate of gliomas has been on the increase. Due to the complexity and high heterogeneity of gliomas, some targeted therapies have limited effectiveness 2. Currently, there are no effective diagnostic and therapeutic options available 3. Therefore, there is an urgent need to develop effective methods for diagnosing and treating gliomas. Glioma-specific biomarkers would offer great therapeutic and prognostic significance. In addition, novel effective molecular markers would help improve the survival time and the quality of life. This study explored the prognostic role of seven biomarkers related to radiosensitivity and ferroptosis.

Radiation therapy (RT) plays a significant role in treating malignant glioblastoma (GBM) 4. Several treatment modalities are used in combination with radiotherapy to improve the survival rate. However, the effects are not satisfactory. In 2005, the landmark EORTC/NCIC trial reported that a combination of temozolomide (TMZ) with radiotherapy could effectively treat gliomas 5. So far, this combination is considered the most effective treatment for gliomas. However, the combination does not have a complete curative effect. Therefore, research is needed to investigate the response to radiotherapy between normal brain tissues and glioma tissues. David et al. reported that glioma cells with wild-type p53 gene were more sensitive to radiation 6. In addition, the inhibition of EGFR gene expression in glioma cells would result in enhanced killing by radiation 7. Therefore, it is essential to identify the radiosensitivity-related biomarkers to guide the evaluation of prognosis in glioma patients.

Recent studies reveal that radiosensitivity is associated with ferroptosis. Gan et al. reported that radiation resulted in the production of large amounts of ROS, up-regulated the expression of ACSL4, (a key ferroptosis enzyme), and increased lipid peroxidation. Further, knockout of the ACSL4 gene in tumor cells was associated with radiation resistance 8. Zhou et al. reported that radiotherapy could promote lipid oxidation and ferroptosis in tumors by inhibiting SLC7A11 9. Stockwell et al. reported that ferroptosis could promote radiation-induced cancer cell death. Ferroptosis activators were shown to increase the sensitivity of tumor cells to radiation 10. Radiotherapy is often combined with chemotherapy or immunotherapy. Recent evidence reveals that ferroptosis is also associated with the efficacy of chemotherapy or immunotherapies 11. Further, ferroptosis activators play essential roles in radiosensitivity and immunotherapy. Targeting ferroptosis could help overcome radiation resistance in glioma cells 12. Therefore, this study explored the clinical relevance and prognostic significance of radiosensitivity and ferroptosis-associated biomarkers in improving the therapeutic effect in gliomas.

In this study, the mRNA sequencing data obtained from the TCGA database was analyzed to determine the prognostic role and expression profile of the differentially expressed genes associated with radiosensitivity and ferroptosis. Further, the intersecting genes were analyzed to construct a prognostic model. Secondly, the results were validated in the Chinese Glioma Genome Atlas (CGGA) cohort. Finally, functional enrichment analysis of the related genes was conducted. This study aims to provide a prognostic model for gliomas.

## Materials and methods

A flowchart of this study is shown in **Supplementary Figure 1**.

### Acquisition of data and the datasets

The RNA sequencing data obtained from the TCGA database, including 169 tumor tissues and five normal tissues, was used as the training cohort (http://cancergenome.nih.gov/) 13. Patients without survival time were excluded and the samples considered in this study were shown in **Supplementary Table 1**. Further, the RNA sequencing data (mRNAseq_693) and relevant clinical data obtained from the CGGA database was used as the validation cohort (http://www.cgga.org.cn/)^14^. Moreover, 395 radiosensitivity associated genes were downloaded from the dbCRSR (http://bioinfo.ahu.edu.cn:8080/dbCRSR/) 15 **(Supplementary Table 2)**. Finally, 259 ferroptosis-associated genes were obtained from the FerrDb data set (http://www.zhounan.org/ferrdb/) 16 **(Supplementary Table 3)**.

### Identification of the differentially expressed genes

Normal brain tissues and glioma tissues were analyzed using the limma package in R software (version 3.6.3). A false discovery rate (FDR) < 0.05 was set for screening DEGs. Univariate cox analysis was used to determine the overall survival (OS). Thirty-six radiosensitive and nineteen ferroptosis-associated DEGs were shown to have a prognostic value. A Veen diagram was then constructed to show the intersection genes. Further, the STRING online platform was used to analyze the interaction between the key genes (https://string-db.org/)^17^. A visual PPI network was constructed using Cytoscape (v3.7.0) 18, 19.

### Construction and validation of the prognostic model

The radiosensitive and ferroptosis-associated DEGs were explored to obtain seven intersecting genes. After that, the seven-gene signature was used to construct the prognostic model using the LASSO Cox regression analysis. The patients were divided into low- and high-risk groups based on the median risk score. The overall survival (OS) time between the two groups was compared using Kaplan-Meier analysis. The FactoMineR R package was used to perform PCA of the signatures. The ROC analysis was used to evaluate the predictive accuracy and risk scores of each gene. Further, nomograms were constructed for predicting the 1-, 2- and 3-year overall survival of the prognostic model. Lastly, the CGGA cohort was used to validate the seven gene model.

### Independent prognostic analysis

Clinical information of the patients (age, gender, stage, IDH, and MGMT) was extracted from the TCGA and CGGA databases. Univariate and multivariate Cox regression analyses were used to analyze these variables in the regression model.

### Functional enrichment analysis

Patients were stratified into low- and high-risk subgroups based on the median risk score. The specific criteria (|log2FC| ≥ 1.5 and FDR < 0.05) were used to filter the DEGs in the two groups. Further, the clusterProfiler R package was used to perform GO and KEGG analyses. Finally, the ssGSEA of the gsva R package was used to determine the infiltrating score of immune cells and estimate the immune-related pathways 20.

### Cell culture

U87 and U251 glioma cells were bought from the cell bank of the Chinese Academy of Sciences. The cells were maintained in DMEM (Gibco, USA) supplemented with 10% FBS at 37 ℃ in a humidified incubator with 5% CO_2_.

### CCK-8 assay and Colony formation assay

The cells were seeded onto 96-well plates at a concentration of 5×10^3^ cells/mL^21^. After incubation for 10 h with 10 uM Erastin (Catalog No. S7242), the cells were exposed to X-rays from 0 to 8 Gy and further cultured for another 24 h. Further, the CCK-8 kit (Beyotime, China) was used to determine cell viability. Absorbance was determined at 450 nm using a Spectrophotometer (Thermo, USA).

The cells were seeded onto six-well plates at a concentration of 1×10^3^ cells/mL 22. After incubation for 10 h with 10 uM Erastin, the cells were exposed to two Gy X-rays, and cultured for another ten days. After that, the cells were fixed using paraformaldehyde. Subsequently, the cells were stained using crystal violet.

### 5-Ethynyl-20-deoxyuridine (EdU) assay

The cells were seeded onto 96-well plates at a concentration of 5×10^3^ cells/mL 23. After incubation for 10 h with 10 uM Erastin, the cells were exposed to two Gy X-rays, and cultured for another 24h. The cells were then incubated with EdU (Beyotime, China) for 2 h, and were fixed with 4% paraformaldehyde and stained sequentially with Apollo dye solution (RiboBio) and DAPI (Invitrogen). The images were captured using a fluorescence microscope (Olympus).

### Statistical analysis

The GraphPad Prism software (Version 8.0.2, CA, USA) was used to to analyze and graph the data in this study. The results are presented as mean ± SD. Differences between the two groups were analyzed by t-tests. A P value <0.05 indicated that the difference was statistically significant.

## Results

### Identification of the prognostic radiosensitivity- and ferroptosis-associated DEGs in TCGA

**Figure 1:**
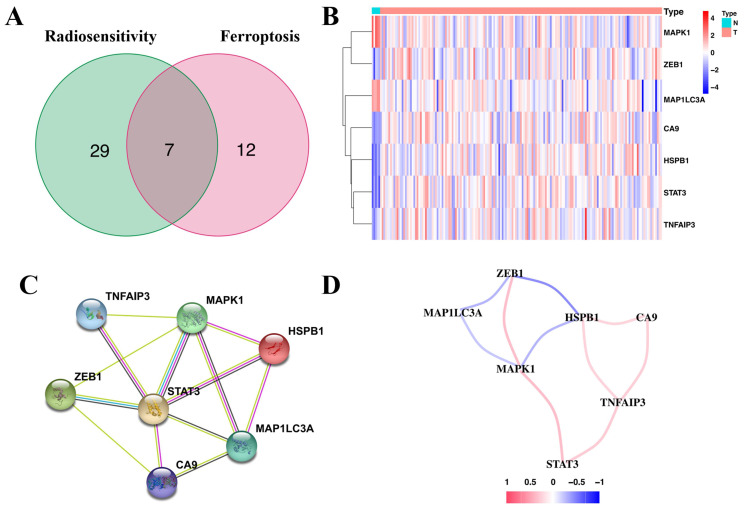
** Identification of the seven-candidate radiosensitivity- and ferroptosis-associated genes in TCGA. (A)** Intersection of the DEGs and the prognostic genes for radiosensitivity and ferroptosis is shown in the veen diagram. **(B)** Five genes were upregulated, while two genes were downregulated in glioma tissues. **(C)** Interactions among the seven candidate genes are shown in the PPI network. **(D)** The red and blue lines represent positive and negative correlations, respectively.

Correlation of the radiosensitivity- and ferroptosis-associated DEGs with OS revealed 36 radiosensitivity- and 19 ferroptosis-associated DEGs with prognostic value **(Supplementary Table 4 and Supplementary Table 5)**. Of these, seven were overlapping genes **(Figure 1A)**. Moreover, ZEB1, CA9, HSPB1, STAT3 and TNFAIP3 of the overlapping genes were upregulated in glioma tissues** (Figure 1B)**. The protein-protein interaction (PPI) network revealed that STAT3 was the hub gene **(Figure 1C)**. The correlation of the overlapping genes is presented in** Figure 1D**.

### Comparison of the expression profile of the overlapping genes between normal brain tissues and glioma tissues

**Figure 2:**
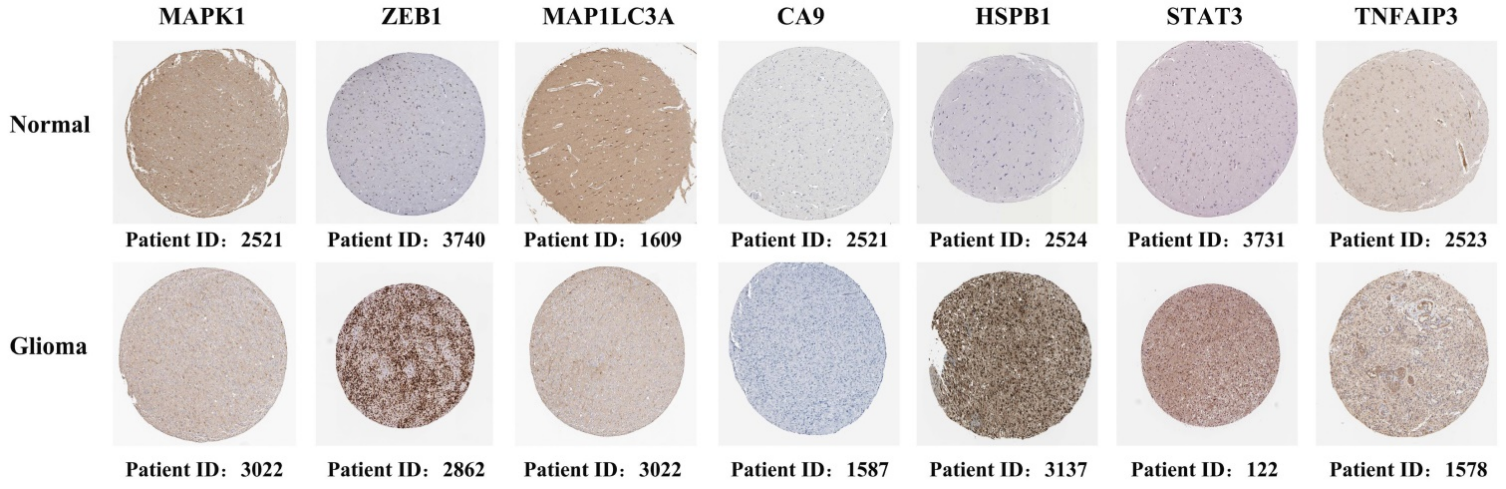
Immunohistochemical staining revealing the expression of the seven candidate genes in normal brain tissues and glioma tissues.

Immunohistochemical staining revealed that five genes (ZEB1, CA9, HSPb1, STAT3, and TNFAIP3) had a higher expression in glioma tissues than in normal tissues. However, MAPK1 and MAP1LC3A had relatively low expression in glioma tissues **(Figure 2)**. These results were consistent with the gene expression patterns in the TCGA database.

### Construction of the prognostic model in TCGA

**Figure 3:**
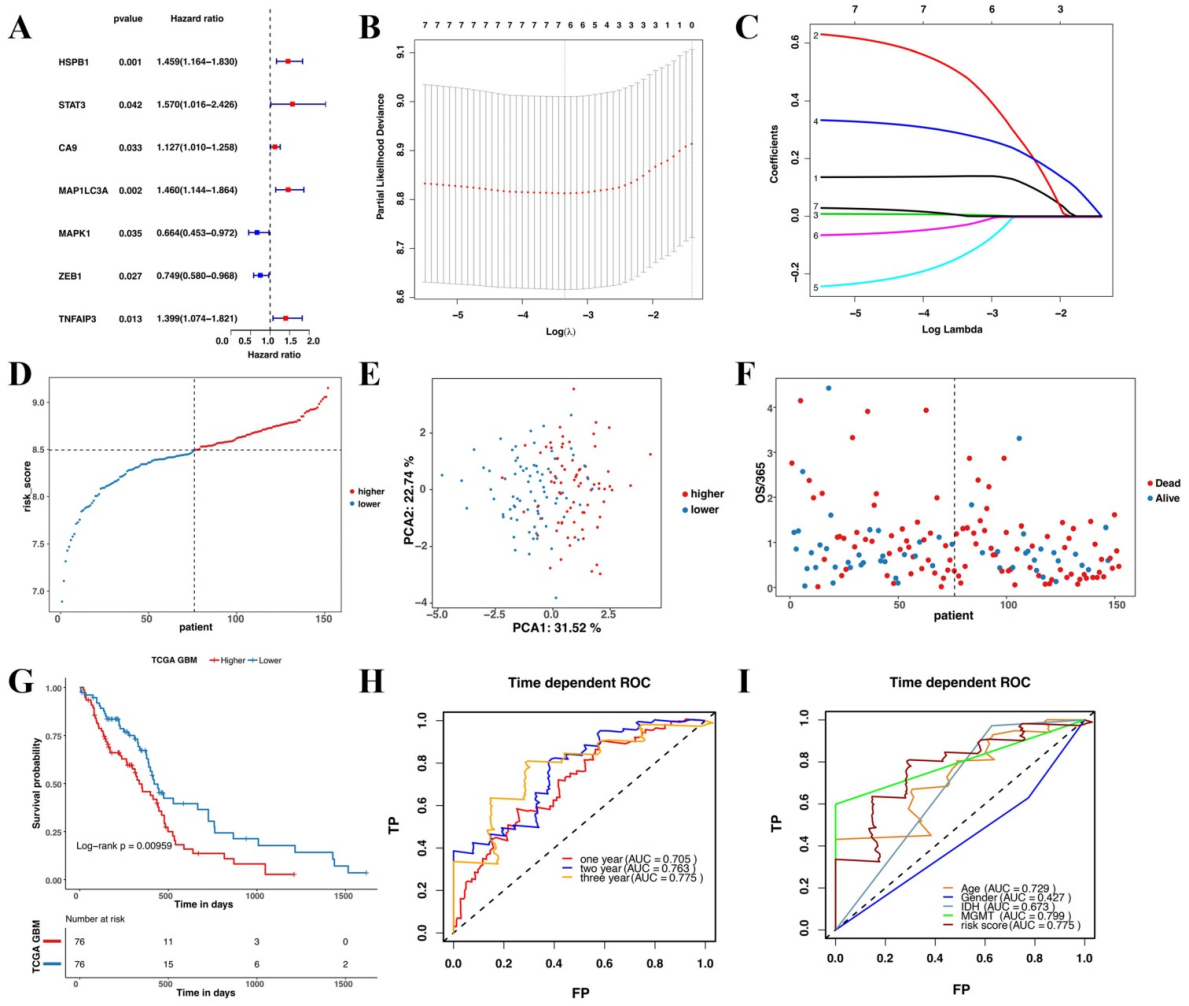
**Construction and evaluation of the prognostic model in TCGA. (A)** Univariate cox regression analysis for each candidate gene. **(B)** Regression coefficients by LASSO of the seven genes. **(C)** The tuning parameters for cross-validation. **(D)** Distribution of the risk scores and the median risk score. **(E)** The PCA plot. **(F)** The survival status for the different groups. **(G)** Kaplan-Meier curves showing survival in the different risk groups. **(H-I)** Area under the receiver operating curves was used to validate the predictive accuracy of the risk signature.

This study revealed seven overlapping genes (MAPK1, ZEB1, MAP1LC3A, HSPB1, CA9, STAT3, and TNFAIP3). Among them, HSPB1, STAT3, CA9, MAP1LC3A, and TNFAIP3 had HRs > 1, suggesting that they were associated with increased risk. The other two genes, MAPK1 and ZEB1 with a HRs < 1 were low-risk genes **(Figure 3A)**. In addition, a molecular signature of the seven genes was constructed using the optimumλvalue to conduct the least absolute shrinkage and selection operator LASSO Cox regression analysis **(Figure 3B and C)**. The risk score was determined as HSPB1*0.139 + STAT3*0.478 + CA9*0.00497 + MAP1LC3A*0.281 + MAPK1*-0.127 + ZEB1*-0.0261 + TNFAIP3*0.000395. Further, the patients were divided into low-risk and high-risk groups based on the median risk score** (Figure 3D)**. Moreover, the patients in the different risk groups were clustered into two groups based on the principal component analysis **(Figure 3E)**. The high-risk group had shorter survival and higher mortality than the low-risk group **(Figure 3F)**. Moreover, overall survival was lower in the high-risk group than in the low-risk group **(Figure 3G)**. Further, ROC analysis was conducted to evaluate the efficiency of the prognostic model. The AUC was 0.705, 0.764, and 0.775 at 1, 2, and 3 years, respectively **(Figure 3H)**. The risk scores for age and MGMT status were 0.729 and 0.799, respectively **(Figure 3I)**.

### Validation of the prognostic model in CGGA

**Figure 4:**
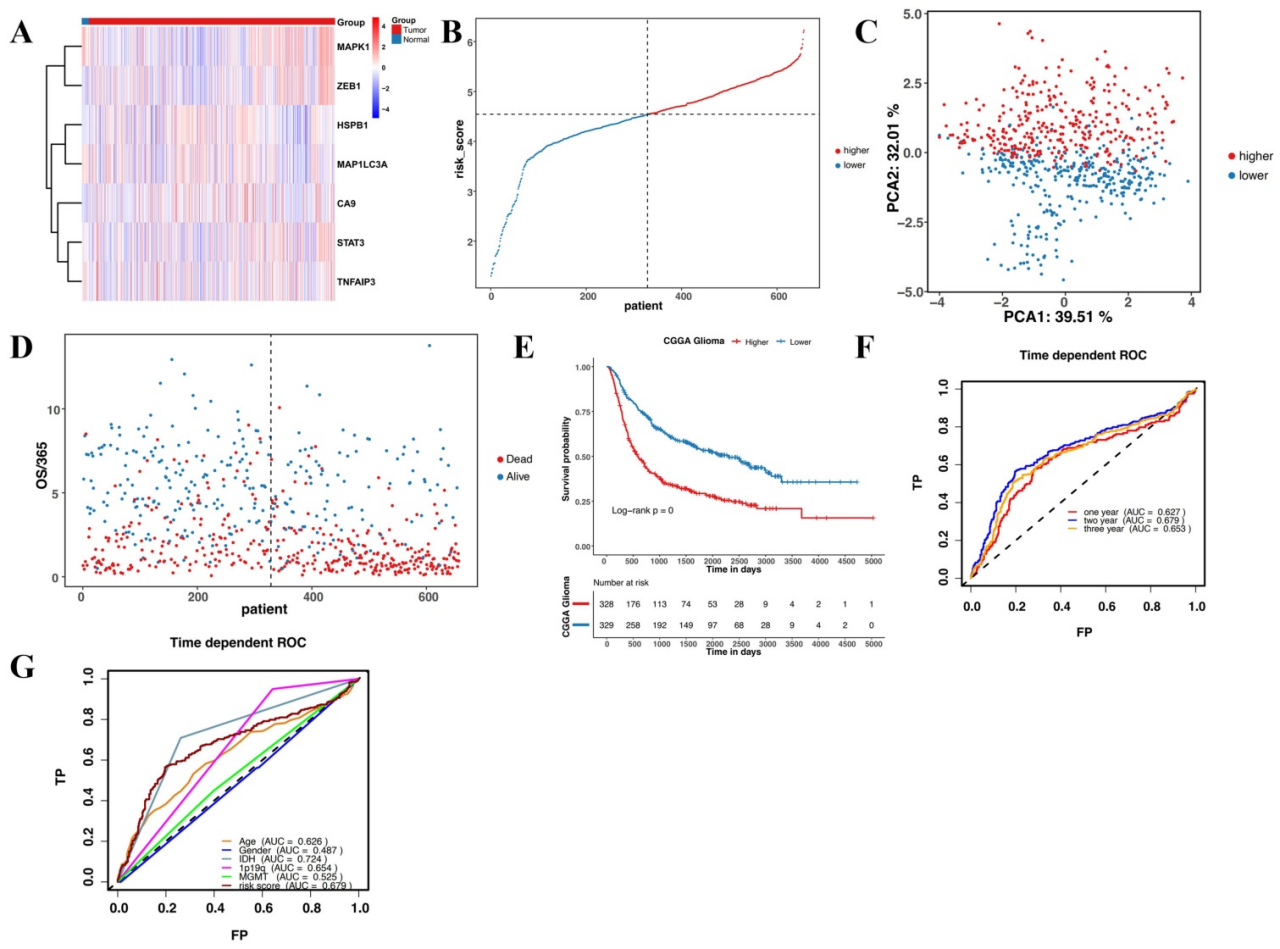
**Validation of the prognostic model in CGGA. (A)** Heatmap of the seven candidate genes in normal brain tissues and glioma tissues. **(B)** Patient distribution based on the median risk score. **(C)** The PCA plot.** (D)** The survival status of patients in different groups. **(E)** Kaplan-Meier curves showing survival in the different risk groups.** (F-G)** Area under the receiver operating curves was used to validate the predictive accuracy of the risk signature.

The validation set to verify the reliability of the prognostic model comprised of 693 glioma patients. Firstly, we detected the expression of the seven genes in CGGA. Gene expression in the CGGA was consistent with the TCGA** (Figure 4A)**. Further, the 693 glioma patients were divided into high-risk and low-risk groups based on the median risk score **(Figure 4B)**. Moreover, the PCA and t-SNE analyses of the CGGA dataset were consistent with the TCGA** (Figure 4C and D)**. Patients in the high-risk group had a poorer prognosis than those in the low-risk group **(Figure 4E)**. The predictive accuracy of the prognostic model in CGGA was further verified in the ROC curve analysis. Results revealed that the seven-gene model had good prognostic accuracy** (Figure 4F and G)**.

### Independent prognostic value of the seven gene risk model

**Figure 5:**
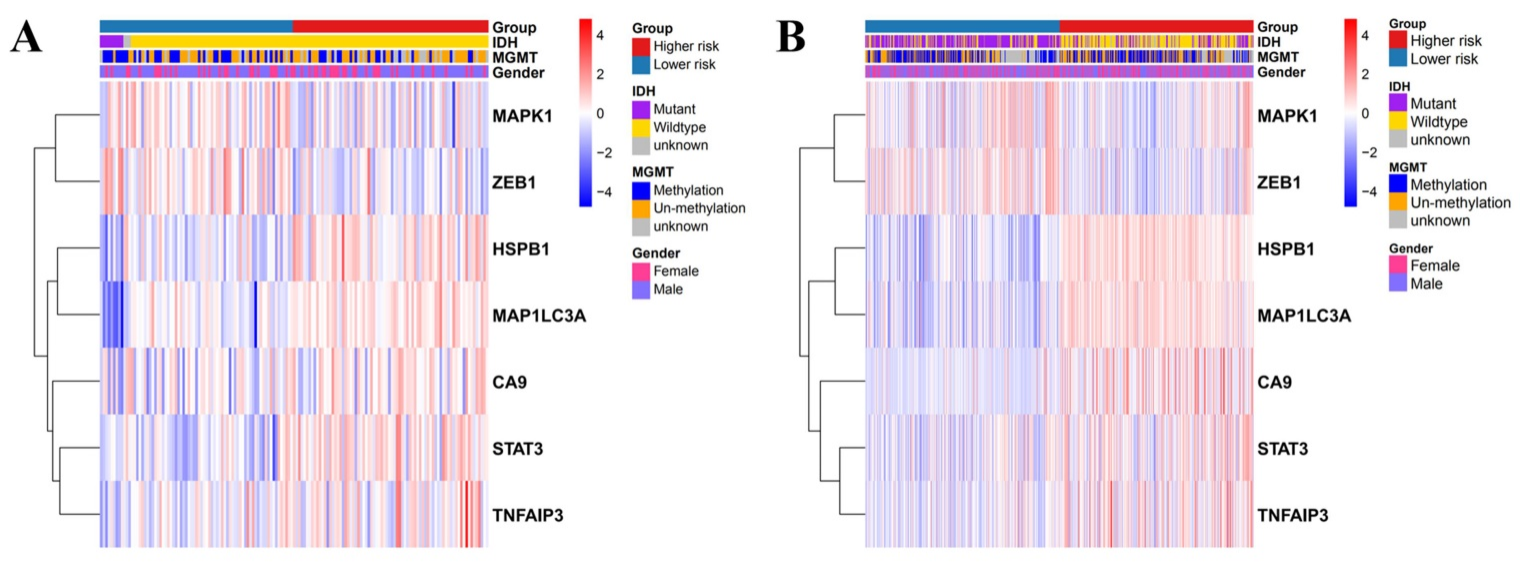
Heatmap showing the association between the clinicopathological features and the risk groups in TCGA (A) and CGGA (B).

Univariate and multivariate Cox regression analyses were used to evaluate the relationship between the risk scores and the clinical parameters (age, gender, IDH, MGMT, and disease staging). Age and disease staging were shown to be independent prognostic predictors for OS in the TCGA and CGGA databases **(Table 1 and 2)**. In the TCGA database, the high-risk genes had a higher expression in the high-risk group, as illustrated by the heatmap in **Figure 5A**. Similarly, the CGGA database revealed similar expression of the high-risk genes **(Figure 5B)**.

### Functional enrichment analyses

**Figure 6:**
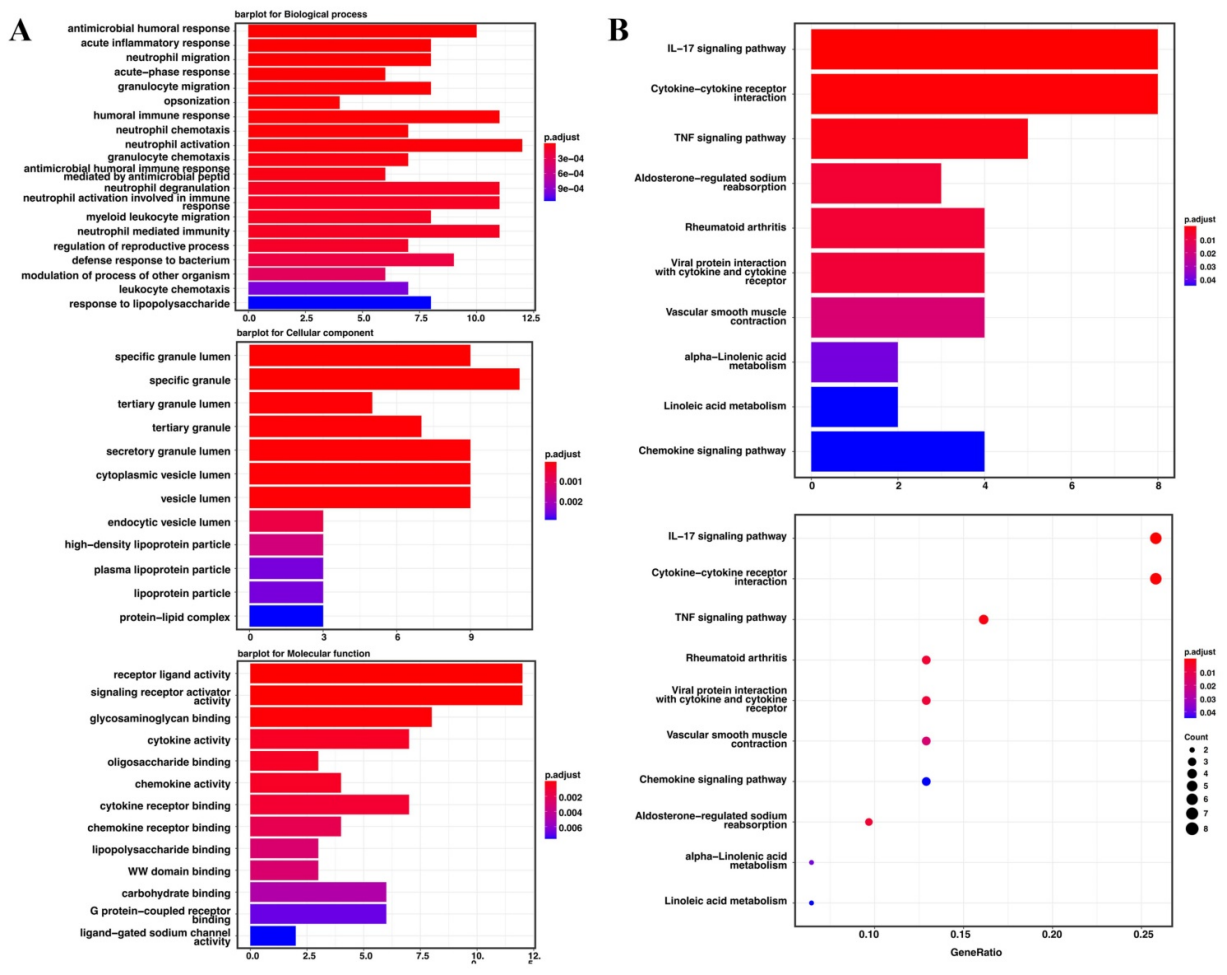
** The Go enrichment analysis and KEGG pathway analysis of the seven candidate genes in TCGA. (A)** Results of the GO enrichment analysis in TCGA. **(B)** Results of the KEGG analysis in TCGA.

Gene ontology enrichment analysis and the KEGG pathway were used to identify the biological characteristics based on the DEGs. The DEGs were shown to be highly expressed in antimicrobial humoral response, acute inflammatory response, neutrophil migration and acute-phase response. The specific granule lumen, specific granule, and tertiary granule lumen were abundant cellular component terminologies. In addition, receptor-ligand activity, signaling receptor activator activity, glycosaminoglycan binding, and cytokine activity were identified as the most abundant functional terms **(Figure 6A)**. The KEGG pathway analysis revealed that the IL-17, cytokine-cytokine receptor interaction, and TNF signaling pathways were the most abundant pathways **(Figure 6B)**.

### Comparison of immune cell infiltration patterns in two different risk groups

**Figure 7:**
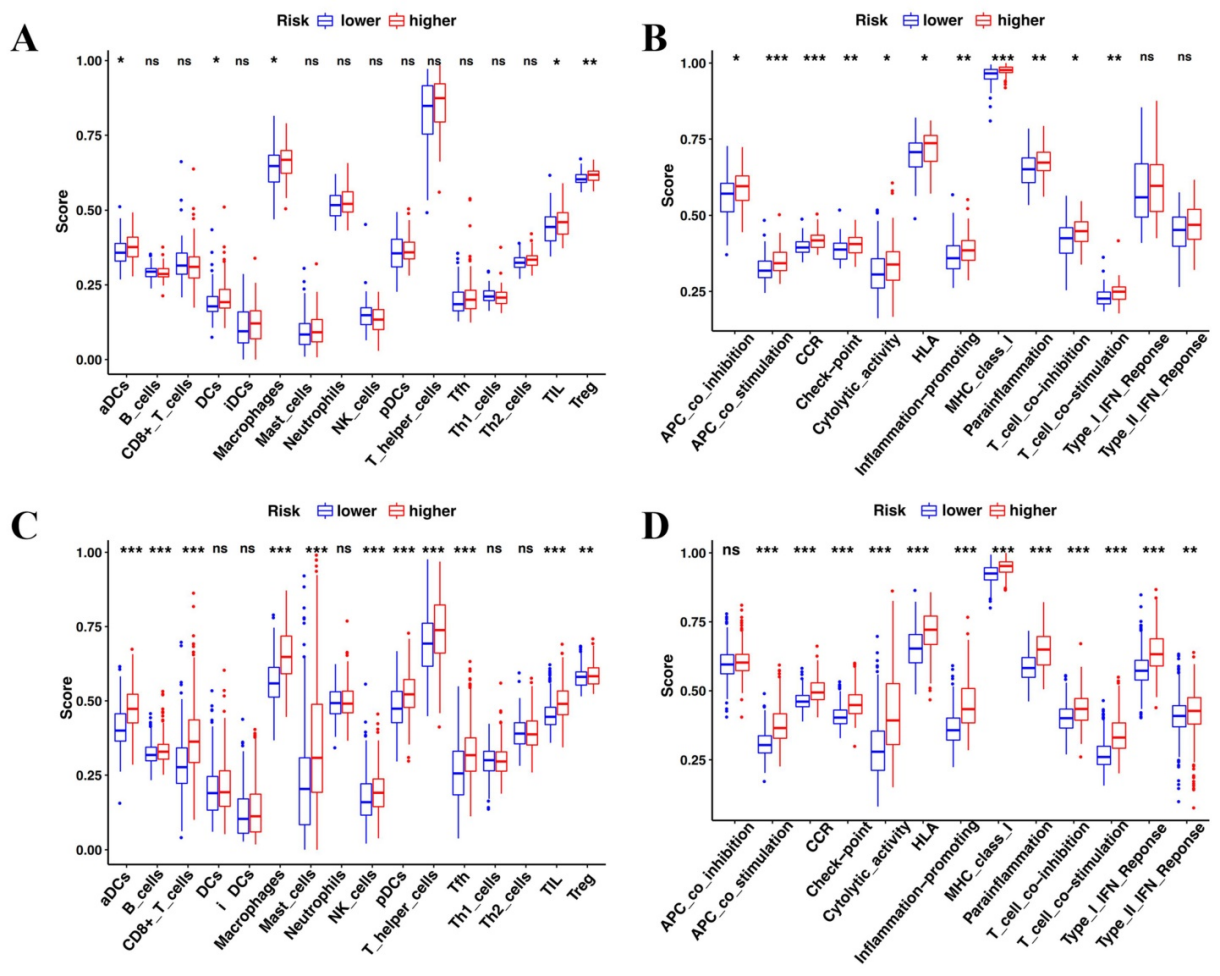
** Comparison of the immune cell infiltration patterns between the high- and low-risk groups. (A-B)** The enrichment scores of 16 types of immune cells and 13 immune-related pathways were compared between the high- and low-risk groups in TCGA. **(C-D)** The enrichment scores of 16 types of immune cells and 13 immune-related pathways were compared between the high- and low-risk groups in CGGA. P-values were shown as: ns, not significant; *P < 0.05; **P < 0.01; ***P < 0.001.

The enrichment analysis revealed that immune features were intrinsically related to radiosensitivity and ferroptosis. Therefore, the immune cell infiltration patterns were compared between the high-risk and low-risk groups by ssGSEA. In the TCGA cohort, aCDs, DCs, Macrophages, TIL, and Treg cells had a higher infiltration in the high-risk group** (Figure 7A)**. Of the 13 immune pathways, the type I IFN and type II IFN response pathways were similar between the high-risk group and the low-risk group in TCGA **(Figure 7B)**. Similarly, the CGGA showed similar immune cell infiltration patterns** (Figure 7C and D)**.

### Association between ferroptosis and radiosensitivity in gliomas

**Figure 8:**
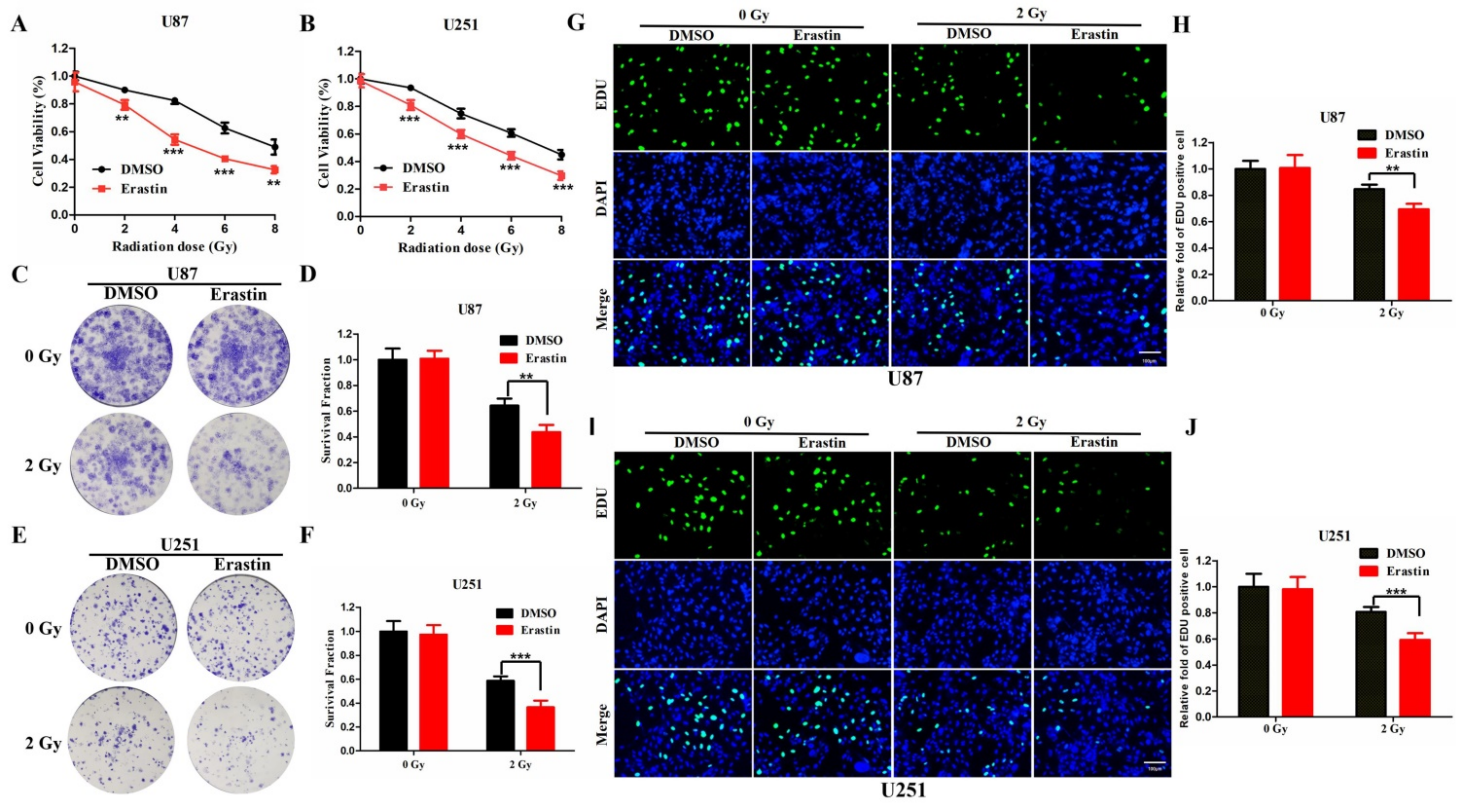
**Erastin-induced ferroptosis enhance the radiosensitivity of glioma cells**. **(A-B)** The CCK-8 cell viability assay following treatment of the U87 and U251 cells with erastin and different radiation doses. **(C-F)** The colony formation assay following treatment of U87 and U251 cells with erastin and radiation. **(G-J)** The EDU assay following treatment of U87 and U251 cells with erastin and radiation. P values were shown as: **P < 0.01, ***P < 0.001.

The ferroptosis inducer, erastin, was used to treat U87 and U251 cells, which were then observed for CCK-8 assay to evaluate the association between radiosensitivity and ferroptosis. Treatment with erastin followed by radiation was shown to significantly inhibit cell viability in the U87 and U251 cells compared to the vehicle (DMSO)-treated cells **(Figure 8A, B)**. Furthermore, the colony formation assay was conducted to evaluate the radiosensitivity of erastin. The erastin-treated U87 and U251 cells were more sensitive to radiation **(Figure 8C-F)**. Similarly, the EDU assay revealed similar conclusions in U87 and U251 cells **(Figure 8G-J).** These results suggested that erastin-induced ferroptosis could enhance the radiosensitivity of glioma cells.

## Discussion

There has been a marked improvement in the diagnosis and treatment of glioma patients. However, gliomas still have high incidence and mortality rates. Radiotherapy is one of the most effective treatments employed in gliomas. Due to high radiation resistance, the therapeutic effects of radiotherapy are unsatisfactory. Several factors contribute to radiation resistance, such as hypoxia, DNA repair, and activation of survival-related signaling pathways. Hypoxia is a key characteristic of the tumor microenvironment, leading to radiation resistance by affecting gene expression 24-26. In addition, radiation can lead to damage of the cellular DNA. The repair process could lead to decreased radiosensitivity or radioresistance of the tumor cells 27, 28. Besides, tumor cells could activate some survival-related signaling pathways following radiation 29-31. Many radiosensitizers have been developed but they cannot meaningfully meet the clinical needs. Therefore, exploring new biomarkers and targets to improve radiosensitivity will help improve the diagnosis, treatment and prognosis of gliomas.

Recent evidence shows that ferroptosis is highly correlated with radiosensitivity. However, a prognostic model of radiosensitivity- and ferroptosis- associated genes evaluating their interaction in gliomas has not been constructed. Therefore, this study conducted a comprehensive analysis of the differential expressed radiosensitivity- and ferroptosis-associated genes. The survival analysis revealed seven prognostic genes (HSPB1, STAT3, CA9, MAP1LC3A, MAPK1, ZEB1, and TNFAIP3). The HSPB1 gene belongs to a conserved protein family which regulates various cellular processes. Zhan et al. reported that HSPB1 could promote cell proliferation in gliomas 32. In addition, HSPB1 regulates extracellular matrix and epithelial-mesenchymal transition, associated with radiation resistance in GBM 33. Furthermore, Tang et al. revealed that HSPB1 was crucial in ferroptosis-mediated cancer therapy 34. STAT3 was associated with poor prognosis in gliomas 35. Bao et al. demonstrated that Ibrutinib could inactivate STAT3, thus reducing radiation resistance in glioma cells. This finding suggested that STAT3 is a risk gene in gliomas, consistent with the results of the present study 36. Hu et al found that bavachin could induce ferroptosis through the STAT3/P53/SLC7A11 Axis 37. Furthermore, CA9 was shown to be highly overexpressed in GBM, conferring radiation resistance in glioma cells 38, 39. Under hypoxia, CA9 could confer resistance to ferroptosis in malignant mesothelioma 40. Michael et al. reported that silencing of MAP1LC3A resulted in significant radiosensitization in lung cancer cells 41. MAPK1 is a tumor promoter in GBM and can be targetted by MicroRNA-362 inhibiting cell growth 42. In addition, ZEB1, a critical regulator of the DNA damage response, is linked to radioresistance^43^. Catherine et al. revealed that α6-integrin could contribute to GBM radioresistance by modulating ZEB1 44. Further, TNFAIP3 was identified as a key ferroptosis-related gene in intracerebral hemorrhage 45. Besides, TNFAIP3 was shown to be a regulator of NFkB in glioma cells and was associated with resistance to O6 alkylating agents 46.

Some preliminary studies show that immune mechanisms play important roles in radiosensitivity and ferroptosis 47-49. Zou et al. revealed that T cells could promote ferroptosis in tumor cells, thus providing a potential therapeutic approach 11. Feng et al. reported that reprogramming the immunosuppressive metabolic microenvironment could improve the curative effect of radiotherapy in breast cancer 50. In this study, the functional enrichment analysis and KEGG pathway analysis revealed that immune cells and immune-related pathways were highly enriched in glioma-related biological processes. These results suggested that the immune microenvironment was highly correlated to radiosensitivity and ferroptosis in gliomas.

## Limitations

However, this study had some limitations. First, this study had a relatively small sample size. Second, this study had a retrospective nature. Therefore, in the future, prospective studies need to be conducted to determine overall survival in follow-up. Third, the results of this study need to be validated in vivo and in vitro experiments. Finally, future studies should explore the specific mechanism related to radiosensitivity and ferroptosis.

## Conclusion

In conclusion, this study is the first to investigate the expression of radiosensitivity- and ferroptosis-associated genes in gliomas. The study identified seven candidate genes and developed a prognostic model. In addition, the study provides a basis for further evaluating the therapeutic role of the seven identified genes.

## Supplementary Material

Supplementary figure and tables.

## Figures and Tables

**Table 1:** Univariate and multivariate Cox regression analyses showing the relationship between the clinicopathological parameters and the prognostic risk model in TCGA

Characteristics	Univariate Cox regression analysis				Multivariate Cox regression analysis			
	HR	HR9.5L	HR9.5H	p Value	HR	HR9.5L	HR9.5H	p Value
Age	1.04	1.02	1.06	<0.001	1.06	1.03	1.1	<0.001
Gender	0.75	0.5	1.13	Ns	0.94	0.55	1.5	0.812
IDH	8.69	2.1	35.94	<0.01	0.74	0.14	4.0	0.723
MGMT	1.63	1.01	2.64	<0.05	0.7	1.21	2.1	0.487
Risk score	3.7	2.02	6.77	<0.01	3.43	1.5	7.9	0.004

**Table 2:** Univariate and multivariate Cox regression analyses showing the relationship between the clinicopathological parameters and the prognostic risk model in CGGA

Characteristics	Univariate Cox regression analysis				Multivariate Cox regression analysis			
	HR	HR9.5L	HR9.5H	p Value	HR	HR9.5L	HR9.5H	p Value
Age	1.03	1.02	1.03	<0.001	1.0	1.00	1.0	0.042
Gender	1.06	0.87	1.3	ns	1.0	0.86	1.3	0.652
IDH	3.09	2.51	3.81	<0.001	1.4	1.01	2.0	0.043
MGMT	1.26	1.01	1.57	<0.05	1.1	0.87	1.4	0.381
Stage								
III	2.55	1.85	3.51	<0.001	2.5	1.84	3.5	<0.001
IV	6.97	5.08	9.56	<0.001	6.1	4.36	8.4	<0.001
Risk score	1.44	1.26	1.66	<0.001	1.2	1.04	1.3	0.01
