# Supplementary material for: Construction of a novel radiosensitivity- and ferroptosis-associated gene signature for prognosis prediction in gliomas
Source: J Cancer. 2022 May 20;13(8):2683–93. doi: 10.7150/jca.72893 (PMC9174846; doi:10.7150/jca.72893)
Supplement: Supplementary file 1 — Supplementary figure and tables. [file jcav13p2683s1.pdf]

Supplementary Figure 1: The specific analysis flowchart of this study.

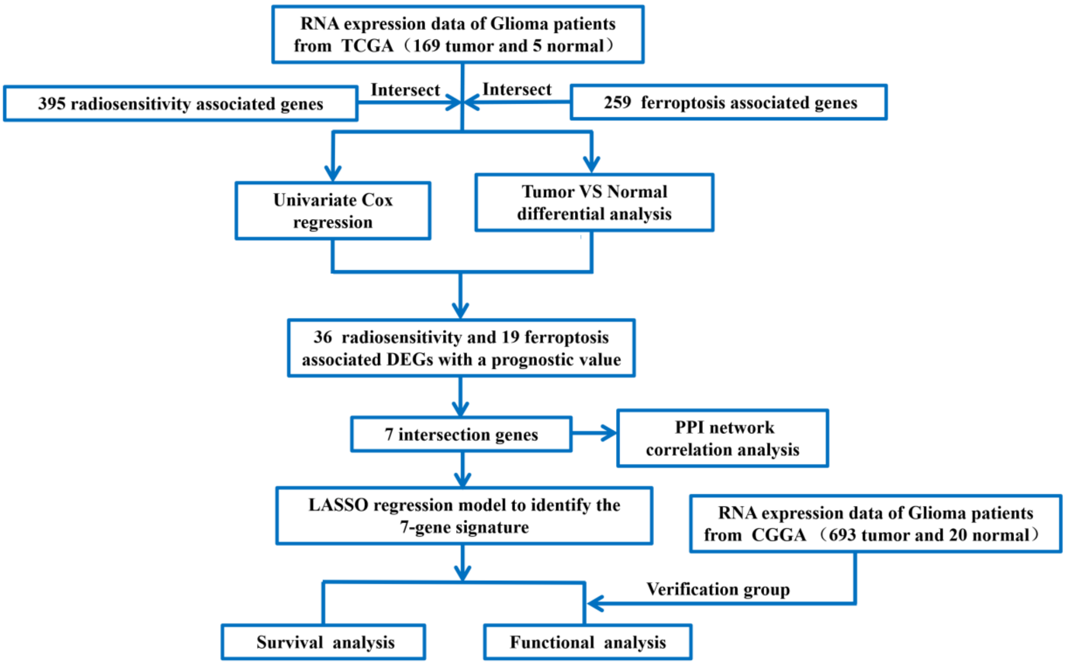

Supplementary Table 1: TCGA samples used in this study.

Sample ID (normal)	Sample ID (GBM)	Sample ID (GBM)	Sample ID (GBM)	Sample ID (GBM)	Sample ID (GBM)
TCGA-06–0675–11	TCGA-02–0047–01	TCGA-06–0646–01	TCGA-06–5858–01	TCGA-19–1787–01	TCGA-28–5204–01
TCGA-06–0678–11	TCGA-02–0055–01	TCGA-06–0649–01	TCGA-06–5859–01	TCGA-19–2619–01	TCGA-28–5207–01
TCGA-06–0680–11	TCGA-02–2483–01	TCGA-06–0686–01	TCGA-08–0386–01	TCGA-19–2620–01	TCGA-28–5208–01
TCGA-06–0681–11	TCGA-02–2485–01	TCGA-06–0743–01	TCGA-12–0616–01	TCGA-19–2624–01	TCGA-28–5209–01
TCGA-06-AABW-11	TCGA-02–2486–01	TCGA-06–0744–01	TCGA-12–0618–01	TCGA-19–2625–01	TCGA-28–5213–01
	TCGA-06–0125–01	TCGA-06–0745–01	TCGA-12–0619–01	TCGA-19–2629–01	TCGA-28–5215–01
	TCGA-06–0125–02	TCGA-06–0747–01	TCGA-12–0821–01	TCGA-19–4065–01	TCGA-28–5216–01
	TCGA-06–0129–01	TCGA-06–0749–01	TCGA-12–1597–01	TCGA-19–4065–02	TCGA-28–5218–01
	TCGA-06–0130–01	TCGA-06–0750–01	TCGA-12–3650–01	TCGA-19–5960–01	TCGA-28–5220–01
	TCGA-06–0132–01	TCGA-06–0878–01	TCGA-12–3652–01	TCGA-26–1442–01	TCGA-32–1970–01
	TCGA-06–0138–01	TCGA-06–0882–01	TCGA-12–3653–01	TCGA-26–5132–01	TCGA-32–1980–01
	TCGA-06-0139-01	TCGA-06–1804–01	TCGA-12–5295–01	TCGA-26–5133–01	TCGA-32–1982–01
	TCGA-06–0141–01	TCGA-06–2557–01	TCGA-12–5299–01	TCGA-26–5134–01	TCGA-32–2615–01
	TCGA-06–0152–02	TCGA-06–2558–01	TCGA-14–0736–02	TCGA-26–5135–01	TCGA-32–2616–01
	TCGA-06–0156–01	TCGA-06–2559–01	TCGA-14–0781–01	TCGA-26–5136–01	TCGA-32–2632–01
	TCGA-06–0157–01	TCGA-06–2561–01	TCGA-14–0787–01	TCGA-26–5139–01	TCGA-32–2634–01
	TCGA-06–0158–01	TCGA-06–2562–01	TCGA-14–0789–01	TCGA-27–1830–01	TCGA-32–2638–01
	TCGA-06–0168–01	TCGA-06–2563–01	TCGA-14–0790–01	TCGA-27–1831–01	TCGA-32–4213–01

TCGA-06–0171–02	TCGA-06–2564–01	TCGA-14–0817–01	TCGA-27–1832–01	TCGA-32–5222–01
TCGA-06–0174–01	TCGA-06–2565–01	TCGA-14–0871–01	TCGA-27–1834–01	TCGA-41–2571–01
TCGA-06–0178–01	TCGA-06–2567–01	TCGA-14–1034–01	TCGA-27–1835–01	TCGA-41–2572–01
TCGA-06–0184–01	TCGA-06–2569–01	TCGA-14–1034–02	TCGA-27–1837–01	TCGA-41–3915–01
TCGA-06–0187–01	TCGA-06–2570–01	TCGA-14–1402–02	TCGA-27–2519–01	TCGA-41–4097–01
TCGA-06–0190–01	TCGA-06–5408–01	TCGA-14–1823–01	TCGA-27–2521–01	TCGA-41-5651-01
TCGA-06–0190–02	TCGA-06–5410–01	TCGA-14–1825–01	TCGA-27–2523–01	TCGA-76–4925–01
TCGA-06–0210–01	TCGA-06–5411–01	TCGA-14–1829–01	TCGA-27–2524–01	TCGA-76–4926–01
TCGA-06–0210–02	TCGA-06–5412–01	TCGA-14–2554–01	TCGA-27–2526–01	TCGA-76–4927–01
TCGA-06–0211–01	TCGA-06–5413–01	TCGA-15–0742–01	TCGA-27–2528–01	TCGA-76–4928–01
TCGA-06–0211–02	TCGA-06–5414–01	TCGA-15–1444–01	TCGA-28–1747–01	TCGA-76–4929–01
TCGA-06–0219–01	TCGA-06–5415–01	TCGA-16–0846–01	TCGA-28-1753-01	TCGA-76-4931-01
TCGA-06–0221–02	TCGA-06–5416–01	TCGA-16-1045-01	TCGA-28-2509-01	TCGA-76-4932-01
TCGA-06–0238–01	TCGA-06-5417-01	TCGA-19-0957-02	TCGA-28-2510-01	
TCGA-06-0644-01	TCGA-06-5418-01	TCGA-19–1389–02	TCGA-28–2513–01	
TCGA-06-0645-01	TCGA-06–5856–01	TCGA-19–1390–01	TCGA-28–2514–01	

Supplementary Table 2: 395 radiosensitivity associated genes.

WEE1	CREB5	PDRG1	CD274	GSTP1	BTG2	LIF	PHF13
SOX2	RASSF1	CHEK1	XRCC4	XPO1	JUN	TPP1	SRC
PIK3CA	TXNRD1	CDH1	CDK4	CDK9	EGF	HDAC9	PAG1
CDKN1A	PPIA	PRDX2	ABCB1	ALDH1A1	ANXA1	PARG	SLC22A3
MAPK1	MARCKS	LIG4	NUMB	BTG1	TERT	ETV4	IGF2BP3
TP53	HOXD10	SOD2	ERBB2	FAS	SMARCA4	UNC5D	HSPB1
APAF1	THY1	CKS1B	SNAI2	PINX1	CSNK2A1	ARRB1	ITGB1
SIRT1	PCSK9	TNFAIP3	SKG1	MGMT	PTK2	ATG12	EIF4E
MT-CO2	SIRT7	GDF15	RAC1	BIRC5	STAT3	PRPF4B	UBE2C
C2CD5	EEF2K	TUSC3	CDC25C	PAK1	UBE2N	ATR	HSP90B1
PTEN	IER5	POU5F1	BRCA1	TOB1	FHIT	EPCAM	MKI67
FOXO3	TGFB1	TNFSF10	TP53INP1	P2RX7	WRAP53	ENPP2	SPARC
IGF1	PDCD4	PDK1	COPS5	CXCL8	HAP1	XRCC5	ERN1
IFI30	EIF2AK3	APBB1	TNFRSF10B	ERAP1	TLR1	ERBB3	PIK3CB
MYC	RRM2B	EGR1	XRCC1	BCL2L2	DUSP1	GPLD1	AIFM3
PVT1	PARP1	HK2	RELB	TP53RK	IFRD1	GLS2	MTDH
PTCH1	NFE2L2	PI4KA	UBE2D3	MDC1	SLC16A1	UHRF1	STAT1
XIAP	CCNB1	MAPK8	FXYD3	KDM5B	12-LOX	AXIN1	EDA
CCDC88A	DUSP3	TTK	HIF1	NR2C2	NOB1	B2M	TERC
LRIG1	BCCIP	CDKN2A	VEGFA	NRP1	TLR4	CA9	REG4
NFKBIA	AKT1	MTOR	PKM	LIG1	RELA	PFN1	NEIL2
IGF1R	HIF1A	EIF2AK2	TCIM	PDGFRB	RAB14	IL6	ERP29

TIMP2	SP1	APTX	KEAP1	CCL22	PGK1	RBBP4	DRAM1
EGFR	FANCD2	PPARA	PIM3	PPA1	CDK1	BUB1	HMOX1
CD44	EMP2	EPHA2	SLC2A1	USP1	IAPP	BUB1B	ASPM
COL1A1	MDM2	ENPP1	SERPINE1	BRCC3	CXCR4	TLR9	BIRC7
BRCA2	HPSE	ASS1	PIM1	LIMS1	CD82	IGFBP7	DIABLO
LHX3	RBP1	PEBP1	LDHA	RB1	ATF2	MAP2K1	FAM168A
PSMB8	RPS6KB1	MCAM	PLK1	RNF8	NPM1	MAP2K2	FGFR3
GSK3B	ZEB1	ROGDI	RAD50	CDC27	POLB	WNT2B	ELAVL4
PBK	AR	REG1A	HBP1	NCL	YAP1	ABCC4	CD40
STK11	RAD51	ITGA6	RNF2	RICTOR	SLPI	AKR1C3	JAK2
HMGB1	TWIST1	PRKDC	H2AFX	AURKA	NOX4	IDH2	GADD45A
SSBP1	SIX1	HDAC1	HMMR	AURKB	API5	CDK2	MAP2K7
TPX2	SOD1	PRRX1	GLS	EPHB1	MRE11	ALK	EPHB2
HMBOX1	Bmi1	RHEB	RPA1	TPM1	APPL1	APEX1	PROM1
TPD52	ATF1	SPP1	DLX2	RSPO1	WISP1	LCN2	PLAUR
ATM	CARM1	RBMS1	PTPN6	DUSP2	CTC1	ID1	SOCS3
ESR1	ALDOA	PEBP4	USP9X	LATS1	SENP1	MSH2	PSMD9
CEP164	ELAVL1	DDB2	S100A9	BRAF	MLH1	CFL1	SPHK1
PAX9	CASP2	DNMT1	MAGED1	DNMT3B	FGF2	ARHGDIB	MAD2L1
RPA3	NTRK1	TP53BP1	ROCK1	BORA	Casp3	PPARG	SIAH1
MAPK14	E2F1	RAD51C	PRMT5	PDLIM7	NBN	HMGB2	TNFRSF6B
MGAT5	BCL2	XRCC6	CTSL	BCL2L1	YWHAQ	CHEK2	AGTR1
CREB1	CDKN3	BSG	CCND1	KLHL34	RAD51D	EZH2	ANPEP
MET	CTNNB1	ERCC1	FGFR2	HLTF	XRCC2	MMP9	
SRP72	PTTG1	S100A4	IGFBP3	TERF2	SLC22A18	IL24	
Bax	KLF10	ING4	RHOA	STMN1	FLCN	MAP1LC3A	
MEF2D	BECN1	KDR	BANP	PRDX1	HAS2	HDAC2	
HSP90AA1	EIF4EBP1	TNF	DAB2IP	WWOX	NCOA3	MAPK9	

Supplementary Table 3: 259 ferroptosis associated genes.

SLC7A11	MIR9-2	DUOX1	ATG16L1	ATM	SLC7A5	EIF2AK4
GPX4	MIR9-3	DUOX2	WIPI1	YY1AP1	HERPUD1	TFAP2C
AKR1C1	CBS	G6PD	WIPI2	EGLN2	XBP1	SP1
AKR1C2	ISCU	PGD	SNX4	MIOX	ZNF419	HBA1
AKR1C3	ACSL3	PIK3CA	ATG13	TAZ	KLHL24	NNMT
RB1	OTUB1	FLT3	ULK2	MTDH	TRIB3	PLIN4
HSPB1	CD44	SCP2	SAT1	IDH1	ZFP69B	HIC1
HSF1	LINC00336	ACSL4	EGFR	SIRT1	ATP6V1G2	STMN1
GCLC	BRD4	LPCAT3	MAPK3	FBXW7	VEGFA	RRM2
NFE2L2	PRDX6	NRAS	MAPK1	PANX1	GDF15	CAPG
SQSTM1	MIR17	KRAS	BID	DNAJB6	TUBE1	HNF4A

NQO1	SESN2	HRAS	ZEB1	BACH1	ARRDC3	NGB
HMOX1	NF2	TF	DPP4	LONP1	CEBPG	YWHAE
FTH1	ARNTL	TFRC	CDKN2A	PTGS2	SNORA16A	GABPB1
MUC1	HIF1A	TFR2	PEBP1	DUSP1	RGS4	AURKA
SLC3A2	JUN	SLC38A1	SOCS1	NOS2	BLOC1S5-TXNDC5	MIR4715
MT1G	CA9	SLC1A5	CDO1	NCF2	LOC390705	RIPK1
SLC40A1	TMBIM4	GLS2	MYB	MT3	EIF2S1	PRDX1
CISD1	PLIN2	GOT1	MAPK8	UBC	KIM-1	MIR30B
FANCD2	MIR212	CARS1	MAPK9	ALB	IL6	
FTMT	Fer1HCH	ALOX5	CHAC1	TXNRD1	CXCL2	
HSPA5	AIFM2	KEAP1	MAPK14	SRXN1	RELA	
ATF4	LAMP2	ATG5	LINC00472	GPX2	HSD17B11	
TP53	ZFP36	ATG7	PRKAA2	BNIP3	AGPAT3	
HELLS	PROM2	NCOA4	PRKAA1	OXSR1	SETD1B	
SCD	CHMP5	ALOX12	ELAVL1	SELENOS	FTL	
FADS2	CHMP6	ALOX12B	BAP1	ANGPTL7	MAFG	
SRC	CAV1	ALOX15	ABCC1	DDIT4	IL33	
STAT3	GCH1	ALOX15B	MIR6852	LOC284561	HAMP	
PML	RPL8	ALOXE3	ACVR1B	ASNS	STEAP3	
MTOR	IREB2	PHKG2	TGFBR1	TSC22D3	DRD5	
NFS1	ATP5MC3	ACO1	EPAS1	DDIT3	DRD4	
TP63	CS	G6PDX	HILPDA	JDP2	MAP3K5	
CDKN1A	EMC2	ULK1	IFNG	SLC1A4	SLC2A1	
MIR137	ACSF2	ATG3	ANO6	PCK2	SLC2A3	
ENPP2	NOX1	ATG4D	LPIN1	TXNIP	SLC2A6	
VDAC2	CYBB	BECN1	HMGB1	VLDLR	SLC2A8	
FH	NOX3	MAP1LC3A	TNFAIP3	GPT2	SLC2A12	
CISD2	NOX4	GABARAPL2	TLR4	PSAT1	GLUT13	
MIR9-1	NOX5	GABARAPL1	ATF3	LURAP1L	SLC2A14	

Supplementary Table 4: 36 radiosensitivity associated DEGs with a prognostic value.

gene	HR	HR.95L	HR.95H	pvalue
SOX2	0.793616162	0.647977073	0.97198904	0.025442624
MAPK1	0.664007175	0.453431204	0.972375796	0.035387166
IFI30	1.676220328	1.123334016	2.501228083	0.011422958
SSBP1	1.533935972	1.013841437	2.320835863	0.042864416
RPA3	1.412179439	1.033484809	1.929637233	0.030254894
MARCKS	0.68765636	0.490794821	0.963480562	0.029542957
HOXD10	1.203405536	1.016975545	1.424011512	0.031086005
RBP1	1.21757478	1.063218546	1.394340185	0.004423551
ZEB1	0.749345246	0.580188127	0.967821077	0.027068421

TNFAIP3	1.398516161	1.073971925	1.82113462	0.012785793
SPP1	1.150000909	1.012237867	1.306513156	0.031809156
S100A4	1.194533488	1.033088814	1.381207728	0.016423264
RELB	1.476942376	1.085167857	2.010157938	0.013150018
SERPINE1	1.149380339	1.021239514	1.293599733	0.020974276
LDHA	1.353851784	1.060437536	1.728451316	0.015065239
CTSL	1.418264238	1.118263865	1.798746712	0.003954139
PAK1	1.514232826	1.100050155	2.084360463	0.010933281
NRP1	1.35483434	1.071821942	1.712575585	0.01108127
ANXA1	1.185858449	1.025398045	1.371428654	0.021557138
STAT3	1.570337348	1.016272959	2.426473482	0.042085927
SLC22A18	1.439535095	1.084620555	1.910586407	0.011657082
LIF	1.200296011	1.043527025	1.380616389	0.010569934
ETV4	1.181230967	1.019222639	1.368990978	0.026901823
CA9	1.127206199	1.009806865	1.258254284	0.03285287
RBBP4	0.56415256	0.36545073	0.870891981	0.009766964
APEX1	0.651982026	0.457983228	0.928157487	0.017611156
ID1	0.820206575	0.680222752	0.988997831	0.037909348
MMP9	1.121457695	1.006592462	1.249430538	0.037603974
IL24	1.484817239	1.002910148	2.198284899	0.048327298
MAP1LC3A	1.460095295	1.14361214	1.86416198	0.002393334
HSPB1	1.459122354	1.163627148	1.829656558	0.001066027
DRAM1	1.362493281	1.037893316	1.78861152	0.025890786
CD40	1.431135151	1.076650559	1.902332938	0.01356581
PLAUR	1.528471859	1.240613468	1.883121766	6.74E-05
AGTR1	1.517318137	1.075687965	2.140262237	0.017516625
ANPEP	1.199512215	1.001609292	1.436517778	0.047991559

Supplementary Table 5: 19 ferroptosis associated DEGs with a prognostic value.

gene	HR	HR.95L	HR.95H	pvalue
HSPB1	1.459122354	1.163627148	1.829656558	0.001066027
SLC40A1	0.783245976	0.634792602	0.966416838	0.022693244
HSPA5	1.575137363	1.132555822	2.190671456	0.006942941
STAT3	1.570337348	1.016272959	2.426473482	0.042085927
CA9	1.127206199	1.009806865	1.258254284	0.03285287
ALOX5	1.235240118	1.015048861	1.503196751	0.034937787
ALOXE3	9.909661833	1.387922645	70.7542297	0.022206612
MAP1LC3A	1.460095295	1.14361214	1.86416198	0.002393334
WIPI1	1.54037719	1.101320152	2.154470597	0.011611525
MAPK1	0.664007175	0.453431204	0.972375796	0.035387166
ZEB1	0.749345246	0.580188127	0.967821077	0.027068421
SOCS1	1.265953168	1.043673551	1.535573477	0.016670684

CHAC1	1.386159002	1.018682934	1.886197084	0.037732735
TNFAIP3	1.398516161	1.073971925	1.82113462	0.012785793
NCF2	1.342669415	1.06311574	1.695733673	0.013368514
TXNIP	1.284063789	1.001166306	1.646899026	0.048936554
RGS4	1.225839385	1.055307117	1.423928801	0.007714391
STEAP3	1.34685299	1.144713	1.584688019	0.000332041
SLC2A3	1.27931446	1.056763351	1.548734148	0.011531106
